# Association of Age and Sex With Multi-Modal Cerebral Physiology in Adult Moderate/Severe Traumatic Brain Injury: A Narrative Overview and Future Avenues for Personalized Approaches

**DOI:** 10.3389/fphar.2021.676154

**Published:** 2021-11-24

**Authors:** C. Batson, A. Gomez, A. S. Sainbhi, L. Froese, F. A. Zeiler

**Affiliations:** ^1^ Department of Human Anatomy and Cell Science, Rady Faculty of Health Sciences, University of Manitoba, Winnipeg, MB, Canada; ^2^ Section of Neurosurgery, Department of Surgery, Rady Faculty of Health Sciences, University of Manitoba, Winnipeg, MB, Canada; ^3^ Biomedical Engineering, Faculty of Engineering, University of Manitoba, Winnipeg, MB, Canada; ^4^ Centre on Aging, University of Manitoba, Winnipeg, MB, Canada; ^5^ Division of Anaesthesia, Department of Medicine, Addenbrooke’s Hospital, University of Cambridge, Cambridge, United Kingdom

**Keywords:** aging, cerebral physiology, sex, TBI - traumatic brain injury, traumatic brain injury (craniocerebral trauma)

## Abstract

The impact of age and biological sex on outcome in moderate/severe traumatic brain injury (TBI) has been documented in large cohort studies, with advanced age and male sex linked to worse long-term outcomes. However, the association between age/biological sex and high-frequency continuous multi-modal monitoring (MMM) cerebral physiology is unclear, with only sparing reference made in guidelines and major literature in moderate/severe TBI. In this narrative review, we summarize some of the largest studies associating various high-frequency MMM parameters with age and biological sex in moderate/severe TBI. To start, we present this by highlighting the representative available literature on high-frequency data from Intracranial Pressure (ICP), Cerebral Perfusion Pressure (CPP), Extracellular Brain Tissue Oxygenation (PbtO_2_), Regional Cerebral Oxygen Saturations (rSO_2_), Cerebral Blood Flow (CBF), Cerebral Blood Flow Velocity (CBFV), Cerebrovascular Reactivity (CVR), Cerebral Compensatory Reserve, common Cerebral Microdialysis (CMD) Analytes and their correlation to age and sex in moderate/severe TBI cohorts. Then we present current knowledge gaps in the literature, discuss biological implications of age and sex on cerebrovascular monitoring in TBI and some future avenues for bedside research into the cerebrovascular physiome after TBI.

## Introduction

Bedside care for moderate and severe traumatic brain injury (TBI) has made significant advances in the past few decades (Hutchinson and O’Phelan, 2014; Carney et al., 2017; Hawryluk et al., 2019; Chesnut et al., 2020). There has been adoption of clinical practice guidelines, to standardize the basics of care (Carney et al., 2017; Hawryluk et al., 2019; Chesnut et al., 2020). Furthermore, we have seen the adoption of multi-modal monitoring (MMM) in neurocritical care (Hutchinson and O’Phelan, 2014; Donnelly et al., 2019), with varying application in the TBI population. These devices, both invasive and non-invasive (Zeiler et al., 2020a), provide complementary information regarding cerebral physiology, so as to improve our understanding beyond that of just intracranial pressure (ICP) and cerebral perfusion pressure (CPP). Such MMM in TBI includes: brain tissue oxygen monitoring (PbtO_2_) (Okonkwo et al., 2017; Zeiler et al., 2020a; Chesnut et al., 2020), parenchymal cerebral blood flow (CBF) monitoring (Mathieu et al., 2019a), cerebral blood flow velocity (CBFV) using transcranial Doppler (TCD) ultrasonography (Czosnyka et al., 1996a; Sorrentino et al., 2011; Budohoski et al., 2012; Hutchinson and O’Phelan, 2014; Zeiler et al., 2017a), regional cerebral oxygen saturations through fixed wavelength near infrared spectroscopy (NIRS) (Mathieu et al., 2019b), and serial extracellular chemistry assessments using cerebral microdialysis (CMD) (Timofeev et al., 2011; Hutchinson et al., 2015; Zeiler et al., 2017b; Zeiler et al., 2017c). Many of these devices are still in their infancy regarding application and available literature to support widespread adoption. However, some, such as PbtO_2_, are emerging as promising new continuous techniques with robust associations with clinical outcome (Okonkwo et al., 2017; Chesnut et al., 2020).

Aside from raw physiologic measures from the above listed MMM devices, there is also an emerging literature body regarding derived metrics of cerebral physiology (Czosnyka et al., 1997; Gao et al., 2017; Calviello et al., 2018; Zeiler et al., 2020a). Such methods employ biomedical engineering techniques of high-frequency signal acquisition and processing of raw recorded physiology, to derive new measures of cerebral physiology. The most widespread discussed metrics are those of continuous cerebrovascular reactivity (Czosnyka et al., 1997; Zeiler et al., 2017d; Zeiler et al., 2019a), which facilitate high-frequency real-time assessment of cerebral autoregulation. The exemplar in this instance would be the pressure reactivity index (PRx) (Czosnyka et al., 1997), which is derived from the correlation between slow-wave vasogenic fluctuation in ICP and mean arterial pressure (MAP), though many others exist in the literature (Zeiler et al., 2017a; Zeiler et al., 2017d; Zeiler et al., 2017e). Aside from cerebrovascular reactivity, other derived measures exist, including: cerebral compensatory reserve monitoring (Calviello et al., 2018; Zeiler et al., 2019b) and entropy metrics (Gao et al., 2017; Zeiler et al., 2020b). PRx has received the most attention, with strong associations with long-term outcomes in adult TBI (Czosnyka et al., 1997; Sorrentino et al., 2012; Zeiler et al., 2019c; Bennis et al., 2020; Zeiler et al., 2020b). However, all such derived measures have been shown to carry some degree of prognostic information (Le Roux et al., 2014).

In addition to this array of modalities and derived cerebral physiologic metrics, the data update frequency at the bedside has drastically increased in the past 10 years (Le Roux et al., 2014; Zeiler et al., 2020a). Classically, studies in moderate/severe TBI and therapeutic guidelines (Carney et al., 2017; Hawryluk et al., 2019; Chesnut et al., 2020) have relied on low-frequency cerebral physiologic data (such as ICP and CPP) recorded in bedside nursing charts. Frequency of such recordings often consisted of point measures recorded hourly, neglecting higher frequency changes or secondary injury patterns. High-frequency (i.e., 100 Hz or higher) data streams are being increasingly relied upon for continuous assessments of cerebral physiologic insult burden, using the percent time spent above known critical thresholds to characterize the burden of secondary injury (Güiza et al., 2015; Zeiler et al., 2019a; Zeiler et al., 2020c; Donnelly et al., 2020; Åkerlund et al., 2020). These insult burden measures have been shown in moderate/severe TBI to provide improved prognostic capacity over classical low-frequency point measures or grand mean values of large epochs of time (Güiza et al., 2017; Donnelly et al., 2020; Åkerlund et al., 2020). Further, as derived cerebral physiologic metrics, such as cerebrovascular reactivity measures, are displaying treatment independence (Zeiler et al., 2019d; Froese et al., 2021) and strong associations with long-term global outcome in moderate/severe TBI, it becomes increasingly clear that such data streams are critical for future work in TBI care.

However, despite the promising nature of MMM, and their high-frequency physiologic data streams, in moderate/severe TBI care and monitoring, there exists many gaps in knowledge, which limit their widespread adoption and interpretation. Of particular importance is the impact of aging and sex on MMM metrics. Age in TBI is a well-known independent prognostic factor (Lingsma et al., 2013; Carney et al., 2017; Maas et al., 2017), with advanced age linked to worse outcomes. Similarly, various studies have documented disparity in long-term outcomes based on sex, despite most moderate/severe TBI populations being dominated by males (Zeiler et al., 2019e; Mikolic et al., 2020). Such biologic sex related differences in moderate/severe TBI outcomes have led to exploration into the impact of sex hormones on secondary injury pathways. Progesterone has been explored extensively in both pre-clinical models, demonstrating reduced neural tissue loss in models receiving exogenous supplementation (Clevenger et al., 2018; Khaksari et al., 2018). These potential benefits of progesterone have led to numerous randomized controlled trials in humans on progesterone supplementation in moderate/severe TBI, with recent meta-analyses demonstrating potentially reduced early mortality (risk ratio (RR) = 0.95; 95% CI = 0.42–0.81; *p* = 0.001) and improved early neurological outcomes (RR = 1.51; 95% CI = 1.12–2.02; *p* = 0.007) (Pan et al., 2019), without sustained long-term differences between cohorts (Lu et al., 2016). However, the exact influence of age and sex on specific MMM based cerebral physiologic measures is often buried, or glossed over, in large TBI outcome studies, where only passing reference may be made between age/sex and monitoring data (Czosnyka et al., 2005; Burkhart et al., 2011; Donnelly et al., 2019; Zeiler et al., 2020b). Historically, much of this limited exploration stemmed from low-resolution point-measured physiology data, which failed to lend itself to more complex analysis on insult burden of cerebral physiologic dysfunction. Yet, recently there has been a shift to recording and archiving of higher frequency digital physiologic data (Czosnyka et al., 1994; Howells et al., 2012; Hutchinson and O’Phelan, 2014; Hawryluk et al., 2020), with various studies emerging in the MMM field in TBI. As such, the biological impact of age and sex on such monitoring metrics in TBI deserves re-evaluation.

Knowledge here could improve our ability to prognosticate in TBI, while also potentially provide us with an ability to forecast acute phase physiology based on age and sex profiles. Further, with the undertaking of randomized controlled trials in MMM in TBI, particularly for PbtO_2_ directed therapies (Okonkwo et al., 2017) and cerebrovascular reactivity based individualized CPP targets (Beqiri et al., 2019), understanding the impact of age and sex on this physiology may help us understand potential subgroups of responders/non-responders.

The purpose of this narrative review is to outline some of the major selected studies pertaining to the impact of age and sex on continuous high-frequency MMM based cerebral physiology in moderate/severe TBI. Further, we explore some of the potential biological explanations for discrepancies seen in such physiologic measures, as they relate to cerebrovascular function/dysfunction. Finally, we will provide some suggestions for future directions into researching the link between aging and sex, with high-fidelity MMM of cerebral physiology in moderate/severe TBI. Of note, for many devices the literature is limited in this area, and only a small number of studies have been commented on. This review is by no means an exhaustive categorization of every study exploring an association between MMM devices and age/sex, but instead aims to provide an overview of the prominent literature, or lack thereof, on this topic.

## Standard Monitoring in Traumatic Brain Injury

### Intracranial Pressure and Cerebral Perfusion Pressure

ICP monitoring, through invasive means, forms the crux of current guideline based management of moderate/severe TBI, with a current threshold for treatment of 22 mmHg (Sorrentino et al., 2012; Carney et al., 2017). Similarly, CPP the second element of current TBI care, which is actively targeted to a range of 60–70 mmHg, and is derived based on the simple formula: CPP = MAP – ICP (Carney et al., 2017). Time spent above the defined ICP thresholds, and outside of the suggested target CPP range, have both been documented to be associated with worse long-term outcome in moderate/severe TBI (Güiza et al., 2017; Donnelly et al., 2020). The major studies in these areas with high-frequency data show that older patients tended to have high ICPs and low CPPs and suffer undesirable outcomes (Czosnyka et al., 2005; Czosnyka et al., 2008). Males were predominant in these studies therefore leading in numbers for fatal outcomes.

Exploring age and ICP/CPP in more detail, it is clear that MMM based high-frequency ICP and CPP have not been directly evaluated in association with age and biological sex. Adams et al. compared the mean ICP and CPP of patients in functional survivors and fatal outcome groups (Adams et al., 2017). Patients in the functional survivors group were younger with mean age 38±16 years vs. 45±18 years (*p* < 0.001) for patients in fatal outcomes group (Adams et al., 2017). Patients in the functional survivors group compared to the fatal outcome group, had lower mean ICP and higher mean CPP (15.1±8.2 mmHg vs. 21.0±10.2 mmHg (*p* < 0.001); and 78.5±8.0 mmHg vs. 75.9± 8.3 mmHg (*p* = 0.009), respectively) (Adams et al., 2017). Nourallah et al. compared the mean ICP and CPP of patients in favourable outcome and unfavourable outcome groups as defined by GOS (Nourallah et al., 2018). Patients in the favourable outcome group were younger with mean age of 38.2±17.0 years vs. 43.0±17.0 years (*p* = 0.0091) for patients in unfavourable outcome group. Patients in the favourable outcome group compared to unfavourable outcome group, had lower mean ICP (12.6±4.6 mmHg vs. 15.4±19.5 mmHg, *p* = 0.0039) but no difference in CPP was found (77.6±6.7 mmHg vs. 77.6± 9.9, *p* = 0.31) (Nourallah et al., 2018). Czosnyka et al. showed in a 2005 study that mean ICP slightly decreased with age from 19±12 mmHg to 15±6 mmHg (*p* < 0.004) and mean CPP increased with age from 72±14 mmHg to 79±12 mmHg (*p* < 0.001) (Czosnyka et al., 2005). Though interpretation of these results must be cautioned, given the primary goal of the analysis in the parent manuscripts was outcome association. Many factors confound patient care trajectories and outcomes, such as institutional treatment guidelines/practices and goals of care discussions between health care providers and families. As such, one cannot directly presume that given younger patients had a higher rate of survival, and the survival groups had lower ICP (or higher CPP), that younger patients have lower ICP/higher CPP. Such inferences should be discouraged at this time.

With regards to biological sex, in the study by Sorrentino et al., it is seen that ICP threshold for survival was lower in males compared to females at 22 vs. 23 mmHg (*p* < 0.001), and lower in patients >55 years of age compared to young, at 21 vs. 23 mmHg respectively (*p* = 0.018) (Sorrentino et al., 2012). Furthermore, the CPP threshold for survival in that study was higher in older patients (>55 years) at 75 vs. 70 mmHg in young patients (*p* = 0.047), regardless of sex. The first study by Czosnyka et al. showed that females <50 years had more fatal outcomes than males, with a 29% death rate in females compared to a 17% death rate in males (*p* = 0.026) (Czosnyka et al., 2008).

It must be emphasized that most large studies on high-resolution (i.e., full waveform) ICP/CPP failed to comment on any detailed statistical association between age or biological sex, other than gross groupings of male vs. female, and arbitrary binary age cutoffs. Further to this, the multi-variable covariance patterns for ICP and CPP were not commented on, only simple comparative statistics have been employed. The studies outlined, are the largest representative studies found, but are by no means exhaustive of all studies documenting the link between ICP/CPP and age/sex. Table 1 provides a summary of selected large studies showing the relationship of these parameters with age and sex.

**TABLE 1:** Main representative studies evaluating link between ICP/CPP and PbtO2 with age/biological sex.

References	Monitoring technique	Number of patients	Patient characteristics	Relevant results	Relevant conclusions
ICP					
Czosnyka et al. (2008)	- Intraparenchymal probe (Camino ICP transducer in 12 patients and Codman ICP Microsensors in 566 patients) or via a ventricular drain and an external pressure transducer (34 patients)	612 total patients, 469 with ICP data	- Severe TBI	- Sex: 371 males; 98 females	- There was no notable difference amongst males and females above or below 50 years of age in mean ICP
				- Mean age: females 34 SD 16.5 years	- Females <50 years old had a notably greater rate of fatal outcome at 6 months compared to males
			- Median admission GCS was 6 in both males and females	Males 34 SD 17 years	- 29% mortality was evident in females (N = 344; *p* = 0.026) vs. 17% in males
Adams et al. (2017)	- Intraparenchymal microsensor (Codman)	601 total patients, 556 with ICP data	- Severe TBI	- Sex: based on total cohort of patients (601): 464 males, 137 females	- Mean ICP and SD in Functional Survivors group was 15.1±8.2 mmHg and in the Fatal Outcome group was 21.0±10.2 mmHg, *p* < 0.001
			- Based on total cohort of patients (601): best pre-intubation GCS 3–8 in 435 patients and 9–15 in 166 patients	Based on 556 patients with ICP data: Functional Survivors group: 360 males, 108 females	- Males were predominant in both groups. The Fatal Outcome group had older patients with higher mean ICPs
			- Two groups of patients were monitored	Fatal Outcome group: 68 males, 20 females	
			Based on 556 patients with ICP data: best pre-intubation GCS in Functional Survivors group was 3–8 in 329 patients and 9–15 in 139 patients	- Mean age: based on total cohort of patients (601): 39 SD 17 years	
			In Fatal Outcome group was 3–8 in 71 patients and 9–15 in 17 patients	Based on 556 patients with ICP data: Functional Survivors group: 38±16 years	
				Fatal Outcome group: 45±18 years	
Sorrentino et al. (2012)	- Intraparenchymal transducer (Codman)	459	- Mild to severe TBI	- Sex: 359 males; 100 females	- ICP Threshold for Survival and Favourable Outcome was lower in patients >55 years and higher in patients ≤55 years
				- Median age: 34, IQR 27 years	
			- Median admission GCS was 7 range 3–15, IQR 5	Patients were categorized into two groups	- ICP Threshold for Survival was lower in males and higher in females and for Favourable Outcome was lower in females and higher in males
				- Threshold for Survival was 23 mmHg, *p* < 0.001 and for Favourable Outcome was 22 mmHg, *p* < 0.001 in patients ≤55 years	
			338 patients had GCS ≤8 and 121 patients had a GCS ≥9	- Threshold for Survival was 21 mmHg, *p* = 0.018 and for Favourable Outcome was 18 mmHg, *p* = 0.023 in patients >55 years	- ICP threshold was lower in females and patients >55 years old for Favourable Outcomes suggesting they were more vulnerable to intracranial hypertension
				- Threshold for Survival in females was 23 mmHg, *p* < 0.001 and for Favourable Outcome was 18 mmHg, *p* = 0.004	
				- Threshold for Survival in males and for Favorable Outcome was 22 mmHg, *p* < 0.001	
Czosnyka et al. (2005)	- Intraparenchymal probe (Camino ICP transducer in 12 patients and Codman ICP Microsensors in 346 patients)	358	- Mild to severe TBI	- Sex: 288 males; 60 females	- Study showed that elderly people suffered worse outcomes post TBI
			- Initial GCS scores ranged from 3–15, 20% patients had a GCS score above 8	- Age range: 16–87 years	- Mean ICP decreased with age (r = − 0.14, *p* < 0.01)
Nourallah et al. (2018)	- ICP: Intraparenchymal Strain gauze probe (Codman)	355	- Moderate to severe TBI	- Sex: 271 males; 84 females	- Mean ICP overall was 14.1 SD 7.7 mmHg, in the Favourable Outcome (GOS ≥4) group was 12.6 SD 4.6 mmHg and in the Unfavourable Outcome group (GOS <4) was 15.4 SD 19.5 mmHg, *p* = 0.0039 for both groups
				Two groups of patients were monitored	
			- GCS median was 7 IQR 3–9	In Favourable Outcome group: 133 males, 39 females and in Unfavourable Outcome group: 138 males, 45 females	- Mean ICP and age were higher in the Unfavourable Outcome group and lower in the Favourable Outcome group
				- Mean age: 40.6 SD 17.2 years	
				In Favourable Outcome group: 38.2 SD 17.0 years and in Unfavourable Outcome group: 43.0 SD 17.0 years	
CPP					
Czosnyka et al. (2008)	- ICP: intraparenchymal probe (Camino ICP transducer in 12 patients and Codman ICP Microsensors in 566 patients) or via a ventricular drain and an external pressure transducer (34 patients)	612 total patients, 469 with CPP data	- Severe TBI	- Sex: 371 males; 98 females	- There was no notable difference amongst males and females above or below 50 years of age in mean CPP
				- Mean age: females 34 SD 16.5 years	- Females <50 years old had a notably greater rate of fatal outcome at 6 months compared to males
	- ABP: indwelling arterial catheter in the radial artery		- Median admission GCS was 6 in both males and females	Males 34 SD 17 years	29% mortality was evident in females (N = 344; *p* = 0.026) vs. 17% in males
Adams et al. (2017)	- ICP: intraparenchymal microsensor (Codman)	601 total patients, 556 with CPP data	- Severe TBI	- Sex: based on total cohort of patients (601): 464 males, 137 females	
	- ABP: radial or femoral artery		- Based on total cohort of patients (601): best pre-intubation GCS 3–8 in 435 patients and 9–15 in 166 patients	Based on 556 patients with CPP data: Functional Survivors group: 360 males, 108 females	- Mean CPP plus SD in Functional Survivors group was 78.5±8.0 mmHg and in the Fatal Outcome group was 75.9±8.3 mmHg, *p* = 0.009
			- Two groups of patients were monitored	Fatal Outcome group: 68 males, 20 females	- Males were predominant in both groups
			Based on 556 patients with ICP data: best pre-intubation GCS in Functional Survivors group was 3–8 in 329 patients and 9–15 in 139 patients	- Mean age: based on total cohort of patients (601): 39 SD 17 years	- The fatal outcome group had older patients with lower mean CPPs
			In Fatal Outcome group was 3–8 in 71 patients and 9–15 in 17 patients	Based on 556 patients with CPP data: Functional Survivors group: 38±16 years	
				Fatal Outcome group: 45±18 years	
Sorrentino et al. (2012)	- ICP: Intraparenchymal transducer (Codman)	459	- Mild to severe TBI	- Sex: 359 males; 100 females	- CPP threshold for survival was higher in older patients and lower in younger patients
	- ABP: invasively measured from the radial or dorsalis pedis artery		- Median admission GCS was 7 range 3–15, IQR 5	- Median age: 34, IQR 27 years	- CPP thresholds for survival was 70 mmHg for both males and females
			338 patients had GCS ≤8 and 121 patients had a GCS ≥9	Patients were categorized into two groups	
				- Threshold for Survival and Favourable Outcome was 70 mmHg	
				- Threshold for Survival was 75 mmHg in those >55 years old, *p* = 0.047 and no threshold was found for Favourable Outcome in this age group	
				- Threshold for Survival was 70 mmHg, *p* < 0.001 and for Favourable Outcome was 70 mmHg, *p* = 0.001 in patients ≤55 years	
				- Threshold for Survival in females was 70 mmHg, *p* = 0.021 and none was found for Favourable Outcome	
				- Threshold for Survival in males was 70 mmHg, *p* < 0.001 and for Favorable Outcome was 70 mmHg, *p* = 0.021	
Czosnyka et al. (2005)	- ICP: intraparenchymal probe (Camino ICP transducer in 12 patients and Codman ICP Microsensors in 346 patients)	358	- Mild to severe TBI	- Sex: 288 males; 60 females	- Study showed that elderly people suffered worse outcomes post TBI
	- ABP: obtained invasively		- Initial GCS scores ranged from 3–15, 20% patients had a GCS score above 8	- Age range: 16–87 years	- Mean CPP increased with age (r = 0.19, *p* = 0.0004)
				- A notable negative relationship was seen between GOS score and age; r = - 0.301, *p* < 0.0001	
Nourallah et al. (2018)	- ICP: Intraparenchymal Strain gauze probe (Codman)	355	- Moderate to severe TBI	- Sex: 271 males; 84 females	- Mean CPP overall was 77.5 SD 8.5 mmHg, in the Favourable Outcome (GOS ≥4) group was 77.6 SD 6.7 mmHg and in the Unfavourable Outcome group (GOS <4) was 77.6 SD 9.9 mmHg, *p* = 0.31 for both groups
	- ABP: radial or femoral lines connected to pressure transducers		- Median GCS was 7 IQR 3–9	Two groups of patients were studied: In Favourable Outcome group: 133 males, 39 females and in Unfavourable Outcome group: 138 males, 45 females	- Mean CPP did not show much variation between groups and amongst patients
				- Mean age: 40.6 SD 17.2 years	
				In Favourable Outcome group: 38.2 SD 17.0 years and in Unfavourable Outcome group: 43.0 SD 17.0 years	
PbtO_2_					
Martini et al. (2009)	- ICP: Camino monitor	629	- Severe TBI	- Sex: 465 males; 164 females	- Mean daily PbO_2_ in PbO_2_ group was 24.7±10.2 mmHg and none was recorded for the ICP-Only group
	- PbO_2_ monitor		- Admission GCS ≤8	373 males monitored in ICP-Only group and 92 in PbO_2_ group	- It was noted that patients in PbO_2_ group was younger than those in the ICP-only group
			Data given for males in two monitored groups: Mean GCS in the ICP-Only group was 5.6±2.3 and in the PbO_2_ Monitored group was 5.1±2.2	- Mean age: of patients in PbO_2_ group was 35.7±16.9 years and of patients in ICP-Only group was 40.7±19.6 years	- No significant findings were made regarding sex
Zeiler et al. (2020d)	- ICP: intraparenchymal strain gauge probe (Codman) and parenchymal fiber optic pressure sensor (Camino)	185 total, 47 with PbtO_2_ data	- Severe TBI	- Sex: 141 males; 44 females	- In the group with Mean ICP Below 15 mmHg, PbtO_2_ was 27 mmHg IQR 23.2–33.1 mmHg, *p* = 0.183 and in the group with Mean ICP Above 20 mmHg, lowering of PbtO_2_ to 22.1 mmHg IQR 18.2–26.2 mmHg, *p* = 0.183 was seen
	- PbtO_2_ Licox probe		- Two groups of males were studied based on Mean ICP Below 15 mmHg and Mean ICP Above 20 mmHg: Median admission GCS of 6 IQR 3–7 for the group with Mean ICP Below 15 mmHg and 7 IQR 3–8 for the group with Mean ICP Above 20 mmHg	122 males monitored in the group with Mean ICP Below 15 mmHg, 41 with PbtO_2_ results and 19 in the group with Mean ICP Above 20 mmHg, 6 with PbtO_2_ results	- Older patients were part of the group with high ICP and low PbtO_2_
				- Median age: of patients in Mean ICP Below 15 mmHg group was 51 IQR 31–62.3 years and of patients in Mean ICP Above 20 mmHg group was 54 IQR 35.3–68.3 years	
Stiefel et al. (2005)	- ICP: Camino monitor	53	- Severe TBI	- Sex: 42 males; 11 females	- A mean daily brain tissue PO_2_ of 34.7±12.3 mmHg was recorded
	- PbtO_2_ monitor		- GCS score <8	Two groups were monitored: ICP/CPP-based therapy group and Combined ICP/CPP and Brain Tissue PO_2_-based therapy group – 25 patients (17 males and 8 females) were in the first group and 28 patients (25 males and 3 females) were in the second group	- Patients who underwent ICP/CPP/Brain Tissue PO_2_ directed management were younger than those who underwent ICP/CPP-based therapy
				- Mean age: of patients in the ICP/CPP group was 44±14 years and of patients in the Brain Tissue PO_2_ group was 38±18 years	

% = percentage, ABP, arterial blood pressure; CPP, cerebral perfusion pressure; GCS, glasgow coma scale; GOS, glasgow outcome scale; ICP, intracranial pressure; IQR, interquartile range, mmHg = millimeters of Mercury, PbtO2 = extracellular brain tissue oxygenation, PBO2 = brain tissue oxygen, PO2 = brain tissue oxygen tension, *p* = *p*-value, r = correlation coefficient, SD, standard deviation; TBI, traumatic brain injury.

### Extracellular Brain Tissue Oxygenation – PbtO_2_

PbtO_2_ is measured using an intraparenchymal Clarke electrode, which assesses extracellular diffusible oxygen levels of the brain parenchyma. Threshold by which intervention is currently suggested by guideline-based approaches is 20 mmHg, values below this are linked to poor outcomes (Okonkwo et al., 2017; Chesnut et al., 2020). This association has triggered ongoing phase III trials in TBI. There are few studies in the literature examining the association between age/sex and PbtO_2_ in moderate/severe TBI. Studies examined showed that older patients trended towards having lower PbtO_2_ and tended to suffer undesirable outcomes (Stiefel et al., 2005; Martini et al., 2009; Zeiler et al., 2020d). Though, it must be acknowledged, that all of the identified relationships failed to reach statistical significance, and only showed a potential relationship based on raw differences in mean/median magnitudes. This lack of significance occurred in the absence of adjusting for multiple comparisons, highlighting that no definitive comments on the association between PbtO_2_ and age/sex can truly be made at this time. Based on the Zeiler et al. study, it can be seen that older patients were part of the group with high ICP and low PbtO_2_ (Zeiler et al., 2020d). In this study, median age for patients in the group with mean ICP below 15 mmHg was 51 (IQR: 31–62.3 years) vs. 54 (IQR: 35.3–68.3 years) (*p* = 0.311) in the group with mean ICP above 20 mmHg, though failed to reach significance (Zeiler et al., 2020d). Those patients in the group with mean ICP above 20 mmHg had lower median PbtO_2_ of 22.1 (IQR: 18.2–26.2 mmHg) vs. 27 (IQR: 23.2–33.1 mmHg) (*p* = 0.183) in those with mean ICP below 15 mmHg, though this failed to reach significance. As can be seen from the identified studies, they both made some reference to age in relation to this PbtO_2,_ however it was somewhat vague. Males were predominant in these studies, but no major association was documented between the sexes. Table 1 provides a summary of the available studies showing the relationship of this parameter with age and sex.

## Advanced Cerebral Monitoring in Traumatic Brain Injury

### Regional Cerebral Oxygen Saturations – Near Infrared Spectroscopy Measures

NIRS is a non-invasive technique which utilizes light in the range 700–1,000 nm on the electromagnetic spectrum to monitor frontal cerebral oxy-hemoglobin and deoxy-hemoglobin concentration, as well as regional cerebral oxygen saturations (rSO_2_) (Mathieu et al., 2020). Thresholds for outcome in TBI remain unclear at this time, though data suggests worse cerebral oxygen saturations, and duration of impairment, are linked to worse long-term outcome (Zweifel et al., 2010; Mathieu et al., 2020). Despite a recent systematic review on NIRS in TBI, there are limited studies available documenting the statistical association between age/biological sex and high-frequency NIRS measures in moderate/severe TBI. These limited studies demonstrated that older patients tended to have low rSO_2_ and suffer undesirable outcomes, though this was only a trend which failed to reach statistical significance (Adatia et al., 2018; Adatia et al., 2020). Adatia et al. showed that the younger patients, with a median age of 54 (IQR: 31) years vs. 61 (IQR: 21) years (*p* = 0.38), had the highest mean rSO_2_ of 66 (IQR: 12%) vs. 63 (IQR: 8%) (*p* = 0.13) (Adatia et al., 2018). While in follow-up, Adatia et al. demonstrated that patients without midline shift were older, with mean age of 62±16 years vs. 59±15 years (*p* = 0.36) (Adatia et al., 2020). In this follow-up work, older patients had lower median rSO_2_ of 60.5 (IQR: 45.1–65.2%) compared to younger patients with rSO_2_ values of 64.7 (IQR: 52.0–68.1%) (*p* = 0.39) (Adatia et al., 2020). There was not a clear breakdown of male vs. females. Table 2 provides a summary of selected large studies showing the relationship of this parameter with age and sex.

**TABLE 2:** Main representative studies evaluating link between NIRS, TDF-based CBF and CBFV with age/biological sex.

**References**	**Monitoring technique**	**Number of patients**	**Patient characteristics**	**Relevant results**	**Relevant conclusions**
NIRS					
Adatia et al. (2020)	- NIRS INVOS 5100	104	- Severe brain injury; - GCS ≤8	Sex: 43 females in the group With Midline Shift and 6 females in the group Without Midline Shift	- Mean rSO_2_, median (IQR) in group With Midline shift was 64.7% (52–68.1%) and in group Without Midline Shift was 60.5% (45.1–65.2%), *p* = 0.39 for both groups
			Two groups of females were studied: Mean and SD was 6±3 in the Patients With Midline Shift group and 7±3 in the Patients Without Midline Shift group (controls)	- Mean age: 59±15 years in the group With Midline Shift and 62±16 years in the group Without Midline Shift, *p* = 0.36	- It was noted that patients without midline shift were older and had lower mean rSO_2_
Adatia et al. (2018)	- NIRS INVOS 5100	85 (16 with TBI)	- Severe brain injury; - GCS ≤8	- Sex: females in No Change group = 3, in Increasing group = 4, in Decreasing group = 6 and in Fluctuating group = 27, *p* = 0.40	- Mean rSO_2_ with median IQR in No Change group was 66% (12%), Increasing group was 57% (16%), Decreasing group was 63% (8%) and Fluctuating group was 17% (21%), *p* = 0.13 for all groups
			Data was for females in four different temperature groups: Median (IQR) GCS in No Change group was 3 (3), Increasing and Decreasing group was 7 (2) and Fluctuating group was 7 (4)	- Median and IQR age: in No Change group was 54 IQR 31 years, Increasing group was 59 IQR 23 years, Decreasing group was 61 IQR 21 years and Fluctuating group was 60 IQR 18 years, *p* = 0.38	- Highest rSO_2_ was noted in the youngest patients belonging to the No Change group
CBF					
Dias et al. (2015)	- Parenchymal thermal diffusion probe	18	- Severe TBI	- Sex: 26 females; 16 males	- The 15 patients who were part of the Cerebrovascular Reactivity (CVR) Preserved PRx <0.25 group had mean CBF of 39.0±20.9 ml/100 g/min and the 3 patients who were part of the CVR Impaired PRx >0.25 group had mean CBF of 36.3±22.2 ml/100 g/min, *p* 0.953 for both groups
			- Patients were divided into two groups	- Mean age: 42 SD 16 years	- Patients in the impaired group were noted to be older and had lower CBF
			Median baseline GCS was 6 IQR 3, Median (IQR) in the Cerebrovascular Reactivity (CVR) Preserved PRx <0.25 group was 7 (4) and CVR Impaired PRx >0.25 group was 4 (2)	15 patients with mean age of 40±16 years were part of the Cerebrovascular Reactivity (CVR) Preserved PRx <0.25 group and 3 patients with mean age of 52±11 years were part of the CVR Impaired PRx >0.25 group	
Dickman et al. (1991)	- Cortical thermal diffusion probe	12	- Moderate and severe TBI	- Sex: 8 males; 4 females	- The following are CBF patterns seen in this study: A 25 year old female maintained normal CBF, 3 males and 1 female age range 30–65 years had reduced CBFs, 3 died and one had a vegetative outcome
			- Mean GCS at admission was 6 range 4–12	- Mean age: 31 range 7–65 years	4 males and 3 females age range 7–48 years had elevated CBFs, 5 died, 1 had mild cognitive deficits and 1 was in a persistent coma for 16 months after injury
CBFV					
Czosnyka et al. (2005)	- Doppler Ultrasound	- 358 patients total, 237 had CBFV monitoring	- Mild to severe brain injury	- Sex: 288 males; 60 females	- Blood flow velocity was not dependent on age (*p* = 0.58)
			- GCS scores ranged from 3–15	- Age range: 16–87 years	- No mention was made of a relationship with patient sex
Bouzat et al. (2011)	- Transcranial Doppler (TCD)	98	- Mild to moderate; - GCS 9–15	- Sex: 64 males and 13 females in the group with No SND and 20 males and 1 female in the group with SND	- Mean blood flow velocity (FVm) was lower at 31 range 18–60 cm/s in the SND group as compared to the No SND group where FVm was higher at 49 range 31–80 cm/s, *p* < 0.01 for both groups
			Two groups of patients were studied: Initial GCS score in group with No secondary neurological deterioration (SND) was 14 (9–15) and in the group with SND was 13 (10–15)	- Age and range: 34, 15–84 years in the group with No SND and 46 range 20–80 years in the group with SND, *p* = 0.04	- Patients in the SND were older and had low FVm
Cardim et al. (2020)	- Transcranial Color-Coded Duplex (TCCD)	95	- Severe TBI	- Sex: 70 males; 25 females	- No significant correlation was found between CBFV and age
			- GCS ranged from 3–8	- Age: Patients were older than 18 years and divided into 3 age groups: group 1 was young adults 18–44 years, group 2 was middle-aged adults 45–64 years and group 3 was older adults above 65 years	- Total FVm and median IQR in males across all age groups was 65.67 (58.41–71.67 cm/s) and for females was 71.67 (62.67–78.67 cm/s), *p* ≤ 0.05 for both groups
					- There was a significant variation in FVm between males and females with females FVm higher in each age group compared to males
					- Also, FVm in the older group 3 patients were lowest in both males and females
Hu et al. (2008)	- TCD	30	- Moderate to severe	- Sex: Data provided for two groups of patients: in Brain Injury group – 23 males and 7 females and in the Control group – 8 males and 4 females	- In Brain Injury group mean CBFV on the left was 65.7±33.9 cm/s and in Control group was 56.5±19.8 cm/s
			- Mean GCS was 6 range 3–13	- Mean age: in Brain Injury group was 38±16 years and in the Control group was 43.7±11.9 years	- In Brain Injury group mean CBFV on the right was 62.5±28.2 cm/s and in Control group was 57.4±16.1 cm/s
					- CBFV was higher in the patients than in controls and patients were younger than controls

% = percentage, CBF(s) = cerebral blood flow(s), cm/s = centimeters per second, CVR, cerebrovascular reactivity; FVm, mean blood flow velocity, GCS, glasgow coma scale; IQR, interquartile range, ml/100 g/min = milliliters per 100 g per minute, NIRS, near infrared spectroscopy, *p* = *p*-value, PRx, pressure reactivity index, rSO2 = regional cerebral oxygen saturations, SD, standard deviation; SND, secondary neurological deterioration; TBI, traumatic brain injury; TCD, transcranial Doppler; TCCD, Transcranial Color-Coded Duplex.

The role of age and biologic sex in cerebral oxygen saturation, as measured by NIRS, has been more extensively examined outside the context of TBI. A recent study examined how rSO2 values differed by age and sex in a cohort of 1,616 adults undergoing cardiac interventions. They found that younger patients (18–49 years old) had significantly higher rSO2 values than middle-aged (50–74 years of age; 67% [95%CI 59–74%] vs. 63% [95%CI 56–69%], *p* < 0.001). Similarly, middle-aged individuals had higher cerebral oxygen saturations than elderly patients (>75 years old; 63% [95%CI 56–69] vs. 60% [95%CI 55–66%], *p* < 0.001). Males were also found to have significantly higher rSO2 values than females (65% [95%CI 58–70%] vs. 58% [95%CI 52–63%]) and while males had significantly higher hemoglobin values and were younger in this cohort, the significant effect of biological sex remained following multiple linear regression analysis accounting for these differences (Robu et al., 2020). In contrast, a longitudinal study of 3,110 healthy individuals over the age of 50 found that cerebral oxygenation was lower in males than females, when measured by NIRS. However, cerebral saturation values were found to also decrease with age (Newman et al., 2020).

### Cerebral Blood Flow and Flow Velocities – Thermal Diffusion and Transcranial Doppler Monitoring

Thermal diffusion flowmetry (TDF) is a technique employed via the use of a cortical or parenchymal probe to monitor CBF, by evaluating the power needed to sustain a temperature difference between a proximal and distal thermistor (Rosenthal et al., 2011). Such devices have seen limited use in the moderate/severe TBI literature, given expertise and cost associated with their use (Mathieu et al., 2019a). As such, when objectively evaluating the link between age/biological sex and high-frequency TDF-based CBF, there are only a few studies available (Dickman et al., 1991; Dias et al., 2015). In the small studies evaluating its use in TBI, low CBF measures have been correlated with worse outcomes. Furthermore, these works demonstrated that older patients tended to have low CBF and suffer undesirable outcomes. Dias et al. showed that patients with preserved cerebrovascular reactivity (measured through pressure reactivity index value of <0.25) were younger with mean age of 40±16 years vs. 52±11 years (*p* = 0.173) in those with impaired cerebrovascular reactivity (Dias et al., 2015). In this study, older patients in the impaired cerebrovascular reactivity group had lower mean CBF of 36.3±22.2 ml/100 g/min, compared to 39.0±20.9 ml/100 g/min (*p* = 0.953) in the younger group of patients, though this failed to reach significance (Dias et al., 2015). The second study referenced gave specific patient examples without reporting *p* values (Dickman et al., 1991). No major association was made between the sexes.

Aside from direct CBF measurement, TCD can be utilized to provide surrogate assessment of flow. TCD employs the use of ultrasound waves to insonate the basal cerebral arteries and assess CBFV non-invasively through Doppler frequency shift in the reflected signal, based on blood flow velocity in the insonated artery (Aaslid et al., 1982). Again, as with TDF, the requirement for technical expertise has limited its use in routine TBI monitoring. Low CBFV has been documented to be associated with worse long-term outcome in moderate/severe TBI populations (Czosnyka et al., 1996a; Budohoski et al., 2012; Czosnyka et al., 2005). As with the other monitoring modalities described above, there exists limited data in moderate/severe TBI patients on the association between CBFV and age/biological sex. The main studies have demonstrated that older patients tended to have low CBFV and suffer undesirable outcomes (Czosnyka et al., 2005; Bouzat et al., 2011; Cardim et al., 2020; Hu et al., 2008). This was seen in the study by Bouzat et al., where older vs. younger patients (median age of 46 (IQR: 20–80 years) vs. 34 (IQR: 15–84 years), *p* = 0.04) had lower mean CBFV of 31 (range: 18–60 cm/s) vs. 49 (range: 31–80 cm/s) (*p* < 0.01), respectively (Bouzat et al., 2011). Cardim et al., showed that older males had the lowest median FVm of 63 (IQR: 57.59–74.83 cm/s) vs. 69.33 (IQR: 65.33–72.67 cm/s) for females (*p* ≤ 0.05) (Cardim et al., 2020). Furthermore, Hu et al. described that mean CBFV was higher in patients (e.g., 65.7±33.9 cm/s on left side of brain compared to 56.5±19.8 cm/s on left side of brain for controls) who were younger than in controls (mean age and SD of patients were 38±16 years and controls were 43.7±11.9 years) (Hu et al., 2008). In contrast, Czosnyka et al. commented that CBFV may be independent of age, but required further investigation (Czosnyka et al., 2005). Table 2 provides a summary of large representative studies showing the relationship between TDF-based CBF and TCD-based CBFV, with age and sex. Outside of TBI, trends in FV and PI as measured by TCD have been identified based on age and sex. One study of 1720 healthy participants found that FVm, FVs, and FVd all decreased significantly with age while PI increased with age. Over the entire cohort, FVm, FV,s and FVd were lower in males than females (Bakker et al., 2004). Similarly, in a cohort of 524 healthy subjects, FV in the MCA were found to decrease with age in both males and females. Notably, the rate of decline was significantly greater in females than males, indicating a sex-specific trajectory to the reduction in FV with age (Alwatban et al., 2021).

## Derived Cerebrovascular Metrics in TBI

### Cerebrovascular Reactivity Monitoring – The Pressure Reactivity Index – “PRx”

Cerebrovascular reactivity monitoring refers to the continuous assessment of cerebral autoregulation, through assessing the response in slow-wave vasogenic fluctuations of a measure of CBF/cerebral blood volume (CBV), to changes in a driving pressure for CBF, such as MAP or CPP (Czosnyka et al., 1997; Zeiler et al., 2017d). Pressure reactivity index (PRx) is the most widely described continuous cerebrovascular reactivity measure in TBI monitoring, and is derived from the moving Pearson correlation coefficient between slow-waves of ICP and MAP, with negative values representing “intact” cerebral autoregulation, and positive values denoting “impaired” autoregulation (Czosnyka et al., 1997; Sorrentino et al., 2012). Various single and multi-center studies have documented the strong association between impaired cerebrovascular reactivity and outcomes in moderate/severe TBI (Zeiler et al., 2019a; Donnelly et al., 2019; Bennis et al., 2020). Similarly, PRx is one of the few multi-modal monitoring metrics which has evidence to support its added prognostic value beyond that of ICP, in models adjusting for baseline admission characteristics (Zeiler et al., 2018a; Zeiler et al., 2019a; Bennis et al., 2020). Again, most studies on PRx do not comment directly on the association between cerebrovascular reactivity and age/biological sex. We have highlighted the main and largest such studies on PRx in Table 3.

**TABLE 3:** Main representative studies evaluating link between PRx and cerebral compensatory reserve with age/biological sex.

**References**	**Monitoring technique**	**Number of patients**	**Patient characteristics**	**Relevant results**	**Relevant conclusions**
Cerebrovascular Reactivity					
Czosnyka et al. (2008)	- ICP: intraparenchymal probe (Camino ICP transducer in 12 patients and Codman ICP Microsensors in 566 patients) or via a ventricular drain and an external pressure transducer (34 patients)	612 total patients, 469 with PRx data	- Severe TBI	- Sex: 371 males; 98 females	- There was significantly worse cerebrovascular pressure reactivity in females compared to males below 50 years; PRx in males was 0.044±0.031 and females was 0.11±0.047, *p* < 0.05
	- ABP: indwelling arterial catheter in the radial artery		- Median admission GCS was 6 in both males and females	- Mean age: females 34 SD 16.5 years	- This was not reflected in patients over 50 years
				Males 34 SD 17 years	- Younger patients who showed abnormal PRx of >0.3 had intracranial hypertension (mean ICP >25 mmHg), seen in 60% females and 20% males (*p* < 0.05)
Adams et al. (2017)	- ICP: Intraparenchymal microsensor (Codman)	601 total patients, 556 with PRx data	- Severe TBI	- Sex: based on total cohort of patients (601): 464 males, 137 females	- Mean PRx and SD in Functional Survivors group was 0.05±0.15 a.u. and in the Fatal Outcome group was 0.16±0.21 a.u., *p* < 0.001
	- ABP: radial or femoral artery		- Based on total cohort of patients (601): best preintubation GCS 3–8 in 435 patients and 9–15 in 166 patients	Based on 556 patients with PRx data: Functional Survivors group: 360 males, 108 females	- Males were predominant in both groups
			- Two groups of patients were monitored	Fatal Outcome group: 68 males, 20 females	- The Fatal Outcome group had older patients with higher mean PRx
			- Based on 556 patients with PRx data: best preintubation GCS in Functional Survivors group was 3–8 in 329 patients and 9–15 in 139 patients	- Mean age: based on total cohort of patients (601): 39 SD 17 years	
			In Fatal Outcome group was 3–8 in 71 patients and 9–15 in 17 patients	Based on 556 patients with PRx data: Functional Survivors group: 38±16 years	
				Fatal Outcome group: 45±18 years	
Sorrentino et al. (2012)	- ICP: intraparenchymal transducer (Codman)	459	- Mild to severe TBI	- Sex: 359 males; 100 females	- PRx threshold for survival was higher in males than females and for favorable outcome was higher in females than males
	- ABP: radial or dorsalis pedis artery		- Median admission GCS was 7 range 3–15, IQR 5	- Median age: 34, IQR 27 years	
			338 patients had GCS ≤8 and 121 patients had a GCS ≥9	- PRx survival threshold was 0.25, *p* < 0.001 while favorable outcome threshold was 0.05, *p* < 0.001	
				- No PRx threshold was found for survival or favorable outcome in patients >55 years old	
				- PRx threshold for survival in patients ≤55 years was 0.3, *p* < 0.001 and 0, *p* < 0.001 for favorable outcome	
				- PRx threshold for survival in females was 0.25, 0.3, *p* < 0.001 and for favourable outcome was 0.25, 0.3, *p* = 0.026	
				- PRx threshold for survival in males was 0.3, *p* = 0.002 and 0, *p* < 0.001 for favorable outcome	
Zeiler et al. (2018)	- ICP: Intraparenchymal strain gauge probe (Codman)	358	- Moderate to severe TBI	- Mean/median age with SD/IQR: RAC < −0.05 group was 39.2 (16.7) years and in ≥0.05 group was 50.6 (17.5) years, *p* value <0.0001 for both groups	- A notable difference was seen between age and APACHE scores of those patients below and above index thresholds; increased age and APACHE scores was seen in those above the thresholds
	- ABP: radial or femoral arterial lines attached to pressure transducers		- Two groups of patients were monitored	For RAC < −0.10 group was 38.9 (16.7) years and in ≥ −0.10 group was 49.7 (17.0) years, *p* value <0.0001 for both groups	- Of statistical significance was patient’s age with impaired cerebrovascular reactivity which showed high AUCs and low *p* values
			Mean and median GCS with SD/IQR for RAC < −0.05 group was 7 (4–10) and in ≥ - 0.05 group was 5 (3–8.25)		- The univariate logistic regression analysis showed generally increasing values for AUC and *p* values for age and sex in relation to PRx >0, 0.25 and 0.35
			For RAC < −0.10 group was 7 (4–10) and in ≥ −0.10 group was 6 (3–8.25)		- Advancing age was linked to impaired autoregulation
Czosnyka et al. (2005)	- ICP: intraparenchymal probe (Camino ICP transducer in 12 patients and Codman ICP Microsensors in 346 patients)	358	- Mild to severe TBI	- Sex: 288 males; 60 females	- Study showed that elderly people suffered worse outcomes post TBI
	- ABP: obtained invasively		- Initial GCS scores ranged from 3–15, 20% patients had a GCS score above 8	- Age range: 16–87 years	- PRx showed deterioration of cerebrovascular autoregulation and worsening of outcomes with age r = 0.24, *p* = 0.003
Cerebral Compensatory Reserve					
Zeiler et al. (2018)	- ICP: intraparenchymal strain gauge probe	358	- Moderate to severe TBI	- Sex: 272 males; 86 females	- No difference was noted in mean RAP variables among males and females, *p* > 0.05
	- ABP: radial or femoral arterial lines attached to pressure transducers		- Median admission GCS was 7 IQR 3–9	- Mean age: 40.6 SD 17.2 years	- There was no relationship between RAP variables and patient age, *p* > 0.05
Zeiler et al. (2019)	- ICP: Intraparenchymal strain gauge probe (Codman), parenchymal fiber optic pressure sensor or external ventricular drain	196	- Moderate to severe TBI	- Sex: 150 males; 46 females	- Mean/median RAP (+/- SD/IQR) was 0.614 (0.206) a.u. and wICP was 5.8 (7.9) mmHg
	- ABP: radial or femoral arterial lines attached to pressure transducers		- Mean/median and SD or IQR for admission GCS was 8±5–13	- Mean/median age: 46.6 SD 19.7 years	- High mean age and compensatory-reserve-weighted intracranial pressure (wICP) was associated with worse outcomes and this considerable difference was noted between both Alive/Dead and Favorable/Unfavorable outcome groups
					- *p* < 0.0001 for high mean age in Alive/Dead group and *p* = 0.001 for Favourable/Unfavourable outcome group
					- *p* < 0.0001 for wICP in Alive/Dead group and *p* = 0.002 for Favourable/Unfavourable outcome group
Czosnyka et al. (1996b)	- ICP: Camino transducer or subdural catheter	56	- Severe TBI	- Sex: 40 males	- There was no overall correlation made about this parameter with age and sex, but three specific examples were given where patients died from uncontrollable intracranial hypertension
	- ABP: radial or dorsalis pedis artery		- Mean GCS was 6 range 3–13	16 females	- In these cases, RAP either dropped from around +1 to 0 or negative values
				- Mean age: 36 range 6–75 years	- Patients: 18 year old, GCS 3 on admission and RAP decreased toward 0 and time average RAP became negative; 35 year old male, GCS 3 on admission, RAP decreased to 0 and 15 year old male, GCS 3, RAP 0

ABP, arterial blood pressure; APACHE, acute physiology and chronic health evaluation; AUC, area under the ROC, curve; ICP, intracranial pressure; GCS, glasgow coma scale; GOS, glasgow outcome scale; IQR, interquartile range, *p* = *p*-value, PRx, pressure reactivity index, % = percentage, ROC, receiver operating characteristic curve, r = correlation coefficient, RAP = (R – correlation, A – pulse amplitude of ICP, P – intracranial pressure), SD, standard deviation; TBI, traumatic brain injury; wICP, compensatory–reserve-weighted intracranial pressure.

**FIGURE 1:**
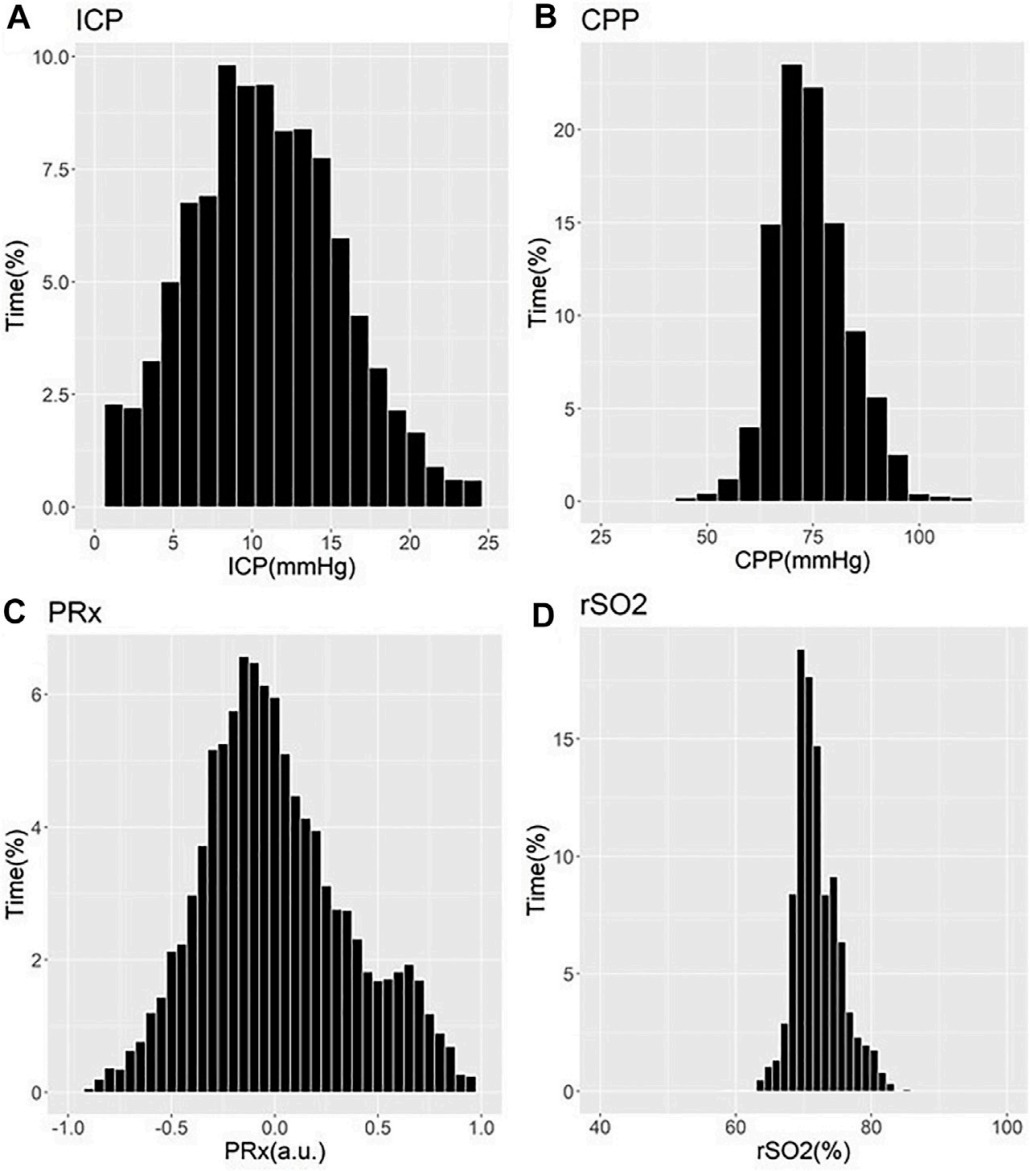
Example of Cerebral Physiology in Severe TBI – 24 Year Old Patient – First 72 Hours of ICU Stay. a. u. = arbitrary units, CPP = cerebral perfusion pressure, ICP = intracranial pressure, ICU = intensive care unit, mmHg = millimeters of Mercury, MAP = mean arterial pressure, PRx = pressure reactivity index (correlation between slow-wave of ICP and MAP), rSO_2_ = regional cerebral oxygen levels (Rt Frontal). Panel **(A)** = histogram of ICP and % time of recorded physiology, Panel **(B)** = histogram of CPP and % time of recorded physiology, Panel **(C)** = histogram of PRx and % time of recorded physiology, Panel **(D)** = histogram of rSO_2_ and % time of recorded physiology.

**FIGURE 2:**
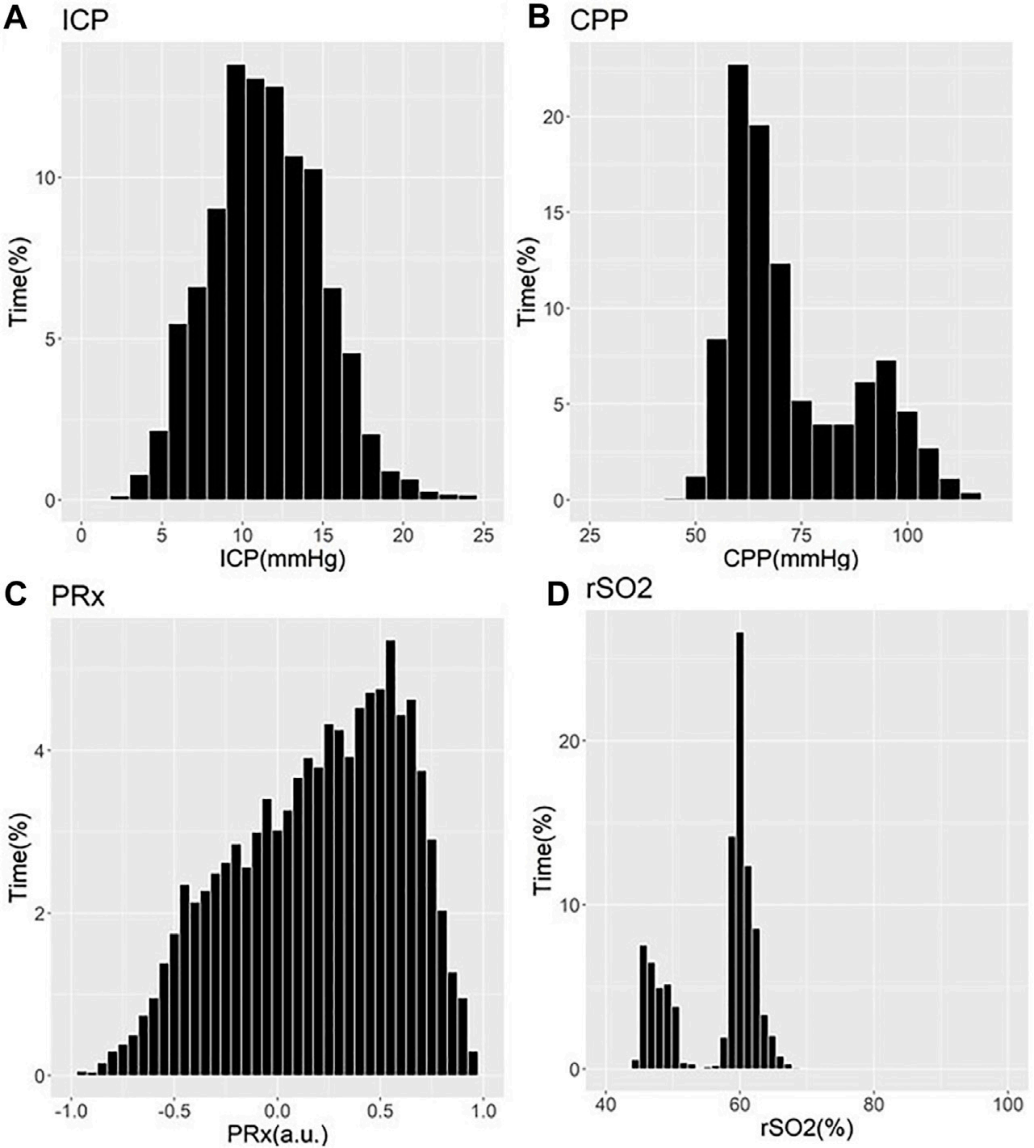
Example of Cerebral Physiology in Severe TBI – 67 Year Old Patient–First 72 Hours of ICU Stay. a. u. = arbitrary units, CPP = cerebral perfusion pressure, ICP = intracranial pressure, ICU = intensive care unit, mmHg = millimeters of Mercury, MAP = mean arterial pressure, PRx = pressure reactivity index (correlation between slow-wave of ICP and MAP), rSO_2_ = regional cerebral oxygen levels (Rt Frontal). Panel **(A)** = histogram of ICP and % time of recorded physiology, Panel **(B)** = histogram of CPP and % time of recorded physiology, Panel **(C)** = histogram of PRx and % time of recorded physiology, Panel **(D)** = histogram of rSO_2_ and % time of recorded physiology.

These works have demonstrated that older patients tended to suffer undesirable outcomes, with trends towards worse cerebrovascular reactivity metrics (Czosnyka et al., 2005; Czosnyka et al., 2008; Sorrentino et al., 2012; Zeiler et al., 2018b; Zeiler et al., 2019a). Three studies showed that older patients with high PRx suffered fatal outcomes. Statistical significance was commented on in two of these PRx studies, one by Czosnyka et al. (2005) and one by Adams et al. (2017) In the Czosnyka et al. (2005) study, it is seen that PRx degraded with increasing age from a mean of 0.01±0.13 a. u. to 0.05±0.12 a. u. (*p* = 0.03) (Czosnyka et al., 2005). In the Adams et al. study, mean PRx of patients in the functional survivors group was lower compared to patients in the fatal outcome group, with results of 0.05±0.15 a. u. vs. 0.16±0.21 a. u. (*p* < 0.001), respectively (Adams et al., 2017). In this study, patients in the functional survivors group were younger with a mean age of 38±16 years vs. patients in the Fatal Outcome group who were older at 45±18 years (*p* < 0.001). Sorrentino et al. found that when evaluating PRx thresholds for survival, PRx was lower in females at 0.25 a. u. vs. males at 0.3 a. u. (*p* = 0.002) (Sorrentino et al., 2012). Further to this, the early study by Czosnyka et al. showed that females <50 years suffered worse cerebrovascular reactivity compared to males, as evidenced by mean PRx for females of 0.11±0.047 a. u. vs. 0.044±0.031 a. u. for males (*p* < 0.05) (Czosnyka et al., 2008). Patients >50 years old in this study did not show comparable differences between males and females (Czosnyka et al., 2008). Of note, we have only commented on those studies on PRx. There exists an extensive and ever-growing literature in moderate/severe TBI on other cerebrovascular reactivity indices, derived from various invasive/non-invasive cerebral monitoring devices (Zeiler et al., 2017d). Figures 1, 2 provide patient examples of high-resolution physiology, documenting differences in ICP, CPP, PRx and rSO_2_ for a young and elderly patient, respectively.

### Compensatory Reserve

Cerebral compensatory reserve monitoring through continuously updating bedside metrics has recently been described in the TBI literature (Kim et al., 2009; Calviello et al., 2018; Zeiler et al., 2018c; Zeiler et al., 2019b). Such measures help with approximating how “tight” or “relaxed” the brain may be post-TBI. RAP (R – correlation, A – pulse amplitude of ICP, P – intracranial pressure) is an index which is derived from the relationship between slow-wave vasogenic fluctuations in ICP and pulse amplitude of ICP (Kim et al., 2009). This gives a constant bedside evaluation of cerebral compensatory reserve where impairment is linked to increased mortality (Calviello et al., 2018; Zeiler et al., 2018c). Further work into RAP is needed in order to employ its use in TBI patients, though preliminary data supports an association between abnormal RAP measures and worse outcomes. As such, there exists a limited literature body documenting the association between RAP and age/biological sex in moderate/severe TBI, with those in existence demonstrating that older patients tended to have undesirable outcomes (Czosnyka et al., 1996b; Zeiler et al., 2018c; Zeiler et al., 2019b). The first study by Zeiler et al. had no difference in mean RAP values between males and females (*p* > 0.05) and no relationship with age (*p* > 0.05) (Zeiler et al., 2018c). The follow-up study by Zeiler et al. demonstrated that increased age and compensatory-reserve-weighted intracranial pressure (wICP) was linked to poor outcomes (Zeiler et al., 2019b). Males were predominant in these studies and no major association was made between the sexes. Table 3 provides a summary of the available RAP literature, documenting any link to age and sex.

## Cerebral Microdialysis – Standard Analytes

CMD is an invasive method of analyzing extracellular fluid for both metabolites and protein biomarkers (Timofeev et al., 2011; Hutchinson et al., 2015; Zeiler et al., 2017b). Classically, CMD is utilized to provide bedside information regarding cerebral metabolites, and typically includes the hourly analysis of: glucose, glycerol, glutamate, lactate and pyruvate. CMD has also been applied to explore for novel biomarkers of injury and physiologic dysfunction (Zeiler et al., 2017c; Helmy et al., 2011a; Guilfoyle et al., 2015). The standard metabolic data obtained from CMD, can be key in guiding clinical therapy and avoiding secondary brain injury and poor outcomes (Timofeev et al., 2011). Though it must be acknowledged, to date CMD use has been limited to a few specialized centers globally, with limited published literature on the topic (Zeiler et al., 2017b). As with all of the described cerebral monitoring devices in moderate/severe TBI, the available literature documenting links between CMD analytes and age/biological sex are limited. Table 4 provides a list of the main available studies documenting such associations with standard CMD analytes. Studies examined generally show that older patients tended to have undesirable outcomes along with those who had low glucose, low pyruvate, high glycerol, high glutamate and high lactate levels, though many of the relationships failed to reach statistical significance (Clausen et al., 2005; Chamoun et al., 2010; Mellergård et al., 2012; Stein et al., 2012; Kurtz et al., 2013). Stein et al. showed that older patients compared to younger patients (median age 50.5 (IQR: 38.3–62.0) vs. 32.5 (IQR: 26.3–45.0 years), respectively; *p* = 0.008) with lower glucose (*p* = 0.061), lower glycerol (*p* = 0.998), higher glutamate (*p* = 0.027), higher lactate (*p* = 0.132) and lower pyruvate (*p* = 0.978) suffered poor outcomes (Stein et al., 2012). Further to this, a study by Mellergard et al. demonstrated that older patients tended to have higher glycerol, higher glutamate, higher lactate and pyruvate (Mellergård et al., 2012). Such patients tended to suffer poor outcomes (Clausen et al., 2005; Chamoun et al., 2010; Mellergård et al., 2012; Stein et al., 2012). Males were predominant in most studies but no comparisons were made between the biological sex categories and CMD analytes.

**TABLE 4:** Main representative studies evaluating link between standard CMD analytes and age/biological sex.

**References**	**CMD analyte**	**Number of patients**	**Patient characteristics**	**Relevant results**	**Relevant conclusions**
Glucose					
Stein et al. (2012)	- Microdialysis catheter CMA 70	89	- Moderate to severe TBI	- Sex: 74 males; 15 females	- Median and IQR range for glucose in the Favourable 6-months Outcome group was 1.3, 0.9–3.1 mmol/L and in the Unfavourable 6-months Outcome group was 0.9, 0.6–1.5 mmol/L, *p* value 0.061 for both groups
	- Perfused using microdialysis pump CMA 106		- Median GCS was 6.5	- Mean age: 46.4 years	- Poor outcomes were seen in older patients and those with lower glucose levels
	- Microdialysis analyzer CMA 600		55 patients had a GCS between 3 and 8 and 34 patients had a GCS 9–12	Median age and IQR in Favourable 6-months Outcome group was 32.5, 26.3–45 years and in the Unfavourable 6-months Outcome group was 50.5, 38.3–62.0 years	
			Two groups of patients were studied: median and IQR GCS in the Favourable Outcome group was 9 (7.3–12.5) and in the Unfavourable Outcome group was 6 (3–10.8)		
Kurtz et al. (2013)	- Microdialysis catheter CMA 70	46	- Moderate to severe brain injury	- Sex: 27 females	- Median glucose was 0.8 IQR 0.4–1.3 mmol/L
	- Perfused using microdialysis pump CMA 106		- Median age was 55 IQR 42–64 years with 27 females and 19 males	19 males	- Levels were low and linked to cerebral metabolic distress and increased mortality, *p* < 0.001
	- Microdialysis analyzer CMA 600		- Median GCS and IQR was 7, 5–9	- Median age: 55 IQR 42–64 years	
Glycerol					
Stein et al. (2012)	- Microdialysis catheter CMA 70	89	- Moderate to severe TBI	- Sex: 74 males; 15 females	- Median and IQR for glycerol in the Favourable 6-months Outcome group was 65.5, 49.1–96.9 μmol/L and in the Unfavourable 6-months Outcome group was 63.7, 42.3–124.6 μmol/L, *p* value 0.998 for both groups
	- Perfused using microdialysis pump CMA 106		- Median GCS was 6.5	- Mean age: 46.4 years	- Poor outcomes were seen in older patients with lower levels
	- Microdialysis analyzer CMA 600		55 patients had a GCS between 3 and 8 and 34 patients had a GCS 9–12; Two groups of patients were studied: median and IQR GCS in the Favourable Outcome group was 9 (7.3–12.5) and in the Unfavourable Outcome group was 6 (3–10.8)	Median age and IQR in Favourable 6-months Outcome group was 32.5, 26.3–45 years and in the Unfavourable 6-months Outcome group was 50.5, 38.3–62.0 years	
Mellergård et al. (2012)	- Microdialysis catheters CMA 71	69	- Severe TBI	- Sex: 48 males; 21 females	- Glycerol in different age groups was studied: in patients <25 years was 63.8±4.40 μmol/L, 25–45 years was 55.9±1.16 μmol/L, 45–65 years was 88.4±2.81 μmol/L and >65 years was 252±15.7 μmol/L, *p* < 0.0001
	- Perfused using microdialysis pump CMA 106		- Glasgow Outcome Scale (GOS) scores: 21 patients with a score of 1, 1 with a score of 2, 11 with a score of 3, 8 with a score of 4, 16 with a score of 5 and 12 with an unknown score	- Mean age: 45.9 years	- Older patients had increased levels and suffered poor outcomes
	- Microdialysis analyzer CMA 600				
Glutamate					
Chamoun et al. (2010)	- Microdialysis probe CMA 70	165	- Severe TBI	- Sex: 141 males; 24 females	- Patients with an average glutamate level >20 μmol/L had a higher mortality rate *p* = 0.08; 76 patients fell in this category
	- Microdialysis analyzer CMA 600		- GCS score ranged from 3–15	- Mean age: 36.6±14.8 years	- There was no correlation between early glutamate levels and age
Stein et al. (2012)	- Microdialysis catheter CMA 70	89	- Moderate to severe TBI	- Sex: 74 males; 15 females	- Median and IQR for glutamate in the Favourable 6-months Outcome group was 3.8, 3.3–6.4 μmol/L and in the Unfavourable 6-months Outcome group was 8.8, 5.8–15.7 μmol/L, *p* value 0.027 for both groups
	- Perfused using microdialysis pump CMA 106		- Median GCS was 6.5	- Mean age: 46.4 years	- Poor outcomes were seen in older patients with higher levels
	- Microdialysis analyzer CMA 600		55 patients had a GCS between 3 and 8 and 34 patients had a GCS 9–12	Median age and IQR in Favourable 6-months Outcome group was 32.5, 26.3–45 years and in the Unfavourable 6-months Outcome group was 50.5, 38.3–62.0 years	
			Two groups of patients were studied: median and IQR GCS in the Favourable Outcome group was 9 (7.3–12.5) and in the Unfavourable Outcome group was 6 (3–10.8)		
Mellergård et al. (2012)	- Microdialysis catheters CMA 71	69	- Severe TBI	- Sex: 48 males; 21 females	- Glutamate in patients <25 years was 21.2±0.94 mmol/L, 25–45 years was 15.8±0.42 mmol/L, 45–65 years was 41.0±2.05 mmol/L and >65 years was 92.2±6.82 mmol/L, *p* < 0.0001
	- Perfused using microdialysis pump CMA 106		- Glasgow Outcome Scale (GOS) scores: 21 patients with a score of 1, 1 with a score of 2, 11 with a score of 3, 8 with a score of 4, 16 with a score of 5 and 12 with an unknown score	- Mean age: 45.9 years	- Older patients had increased levels and suffered poor outcomes
	- Microdialysis analyzer CMA 600				
Lactate and Pyruvate					
Clausen et al. (2005)	- Microdialysis probe CMA 20	151 total, 139 had Microdialysis monitoring	- Severe TBI	- Sex: 113 males	- Lactate**:** Mean lactate of all patients was 976±71 μmol/L, of patients with good outcomes was 785±119 μmol/L and of those with poor outcomes was 1,051±118 μmol/L
	- Microdialysis analyzer CMA 600		- GCS was 3–8 at admission	38 females	- Mean lactate in patients with good outcomes decreased from 951±138 μmol/L to 672±109 μmol/L from day 1 to day 2 respectively and did not really change for the subsequent days resulting in no statistical significance
				- Mean age and SD was 35.5±16.9 years	- Mean lactate in patients with poor outcomes declined steadily from 1,197±111 μmol/L to 718±151 μmol/L respectively from day 1 to day 4, *p* < 0.05
					- Older persons (and majority males) were noted to have high lactate levels and had poor outcomes (died or remained in persistent vegetative state)
Stein et al. (2012)	- Microdialysis catheter CMA 70	89	- Moderate to severe TBI	- Sex: 74 males	- Median and IQR for lactate in the Favourable 6-months Outcome group was 1.8, 1.5–3.0 mmol/L and in the Unfavourable 6-months Outcome group was 2.7, 1.5–4.3 mmol/L, *p* value 0.132 for both groups
	- Perfused using microdialysis pump CMA 106		- Median GCS was 6.5	15 females	- Poor outcomes were seen in older patients with higher levels
	- Microdialysis analyzer CMA 600		55 patients had a GCS between 3 and 8 and 34 patients had a GCS 9–12	- Mean age: 46.4 years	- Median and IQR for pyruvate in the Favourable 6-months Outcome group was 78.7, 51.3–117.5 μmol/L and in the Unfavourable 6-months Outcome group was 77.9, 49.9–121.8 μmol/L, *p* value 0.978
			Two groups of patients were studied: median and IQR GCS in the Favourable Outcome group was 9 (7.3–12.5) and in the Unfavourable Outcome group was 6 (3–10.8)	Median age and IQR in Favourable 6-months Outcome group was 32.5, 26.3–45 years and in the Unfavourable 6-months Outcome group was 50.5, 38.3–62.0 years	- Poor outcomes were seen in older patients with lower levels
Mellergård et al. (2012)	- Microdialysis catheters CMA 71	69	- Severe TBI	- Sex: 48 males	- Lactate in patients <25 years was 5.0±0.06 mmol/L, 25–45 years was 5.0±0.06 mmol/L, 45–65 years was 6.0±0.08 mmol/L and >65 years was 6.3±0.13 mmol/L
	- Perfused using microdialysis pump CMA 106		- Glasgow Outcome Scale (GOS) scores: 21 patients with a score of 1, 1 with a score of 2, 11 with a score of 3, 8 with a score of 4, 16 with a score of 5 and 12 with an unknown score	21 females	- Older patients had increased levels and suffered poor outcomes
	- Microdialysis analyzer CMA 600			- Mean age: 45.9 years	- Pyruvate in patients <25 years was 221±2.55 mmol/L, 25–45 years was 216±2.43 mmol/L, 45–65 years was 256±2.73 mmol/L and >65 years was 227±4.20 mmol/L
					- Older patients had increased levels and suffered poor outcomes
Kurtz et al. (2013)	- Microdialysis catheter CMA 70	46	- Moderate to severe brain injury	- Sex: 27 females	- Median lactate was 3.9 IQR 2.9–4.8 mmol/L
	- Perfused using microdialysis pump CMA 106		- Median age was 55 IQR 42–64 years with 27 females and 19 males	19 males	- Levels were high and linked to cerebral metabolic distress and increased mortality, *p* < 0.001
	- Microdialysis analyzer CMA 600		- Median GCS and IQR was 7, 5–9	- Median age: 55 IQR 42–64 years	- Median pyruvate was 123 IQR 92–160 μmol/L
					- Levels were low and linked to cerebral metabolic distress and increased mortality, *p* < 0.001

% = percentage, CMA, cerebral microdialysis analyzer; CMD, cerebral microdialysis; GCS, glasgow coma scale; GOS, glasgow outcome scale; IQR, interquartile range, mmol/L = millimole per liter, *p* = *p*-value, TBI, traumatic brain injury, r = correlation coefficient, µmol/L = micromole per liter.

## Limitations

As can be seen by the overviewed literature body, the available data is heterogeneous, with significant disparities both within and between monitoring modalities. It must be acknowledged that the goal of our review is not to be exhaustively comprehensive in outlining every article in existence with respect to age/sex and the specific monitoring. However, we did highlight the most representative/pertinent articles, with some of the largest moderate/severe TBI populations, for each high-frequency continuous MMM metric. Our findings do highlight the need for dedicated research on the biological influence of aging and sex on MMM measures, as most of the outlined studies focus on basic associations between chronological age, or binary sex designations, with gross summary metrics for each monitoring variable gained from calculated mean/median over large epochs of monitoring, often over the entire ICU stay. All studies focused mainly on grand averaged data over large epochs of acute phase physiology. Such coarse summary metrics make it near impossible to determine if sub-classes of physiologic profiles exist based on age and sex. This is an important consideration for the design of future studies on the topic.

## Considerations and Future Directions

Our review has highlighted an important gap in knowledge pertaining to MMM in TBI. The existing literature body on the impacts of aging and sex on the cerebral vasculature outside of TBI does provide us some clues as to critical considerations, when planning for future studies on the topic. As much of the MMM based metrics are grounded in cerebrovascular physiologic responses, exploring such non-TBI literature bodies may provide important insights. We will briefly touch on some of these biological factors, but refer the reader to the referenced literature body for more nuanced detail, if desired. Finally, the last subsection outlines some important considerations for future studies moving forward.

### Advanced Age as a Biological Consideration

Advanced age is known to be associated with impaired cerebrovascular response through cerebrovascular reactivity testing in healthy populations during pCO_2_ challenges in concert with advanced functional neuroimaging (McKetton et al., 2018; Miller et al., 2019; Taneja et al., 2020), and TCD assessments of MCA CBFV (Mariappan et al., 2015; Aguilar-Navarro et al., 2019; Klein et al., 2020; Tomoto et al., 2020). This has been confirmed in both populations of awake and anesthetized patients. We have also seen that advanced age is associated with worse cerebrovascular reactivity in moderate/severe TBI patients in our above review of MMM (Czosnyka et al., 2005; Zeiler et al., 2019a; Zeiler et al., 2019c).

There are a variety of pathological changes that could be associated with impaired cerebral vascular performance during the aging process (Camici et al., 2015). First, accumulation of amyloid protein precursor and beta amyloid in the wall of cerebral vessels has long been known to occur with advancing age, as seen in both human literature and experimental models (Grinberg et al., 2012; Weller et al., 2015; Qi and Ma, 2017). Such accumulation has been linked to reduced endothelial nitric oxide (NO) activity secondary to shuttling to the formation of peroxynitrite, and an increase in reactive oxygen species (ROS) (Hamel et al., 2008; Carvalho and Moreira, 2018). This leads to impaired resting tone, as well as attenuated vasodilatory responses of the cerebral vessels. In addition, the general response of the cerebral vasculature to free radicals and ROS becomes impaired. The angiotensin II (AGT) pathway is known to be involved in ROS vascular responses. With advanced aging, it appears that there is an uninhibited angiotensin II mediated generation of ROS, further potentiating the impacts on the NO vasodilatory pathways (Hamel et al., 2008; Camici et al., 2015; Carvalho and Moreira, 2018; Wang et al., 2019).

Aside from NO/ROS mediated changes seen with aging, chronic inflammation has also emerged as a potentially important pathological driver of cerebrovascular dysfunction (Gu et al., 2019; Low et al., 2019; Mészáros et al., 2020).Advanced age has demonstrated increased expression of various systemic pro-inflammatory cytokines, such as interleukin (IL)-1B and IL-6, in both experimental and human literature (Gu et al., 2019). This response is believed to be a maladaptive autoimmune action to cellular breakdown products found during the normal aging process. Furthermore, recent literature also suggests some of the chronic cerebral vascular changes seen with aging, such as amyloid deposition, lead to increased leukocyte adhesion, further potentiating the immune response (Gu et al., 2019; Low et al., 2019; Mészáros et al., 2020). This chronic immune response may be linked to ongoing dysfunction in the central nervous system, and degeneration. Increased circulating IL-6 levels have even been associated with magnetic resonance imaging (MRI) defined white matter ischemic volumes in elderly populations (Gu et al., 2019; Low et al., 2019). The exact mechanism of cerebrovascular dysfunction related to chronic neuroinflammation remains unclear at this time. However, of interest, the acute TBI literature has also found strong associations between serum and cerebrospinal fluid (CSF) pro-inflammatory cytokines, such as IL-6, with poor long-term outcome (Thelin et al., 2017a; Zeiler et al., 2017c; Thelin et al., 2018). Furthermore, the impact of the immune response post-TBI has also been supported by the association between various single nucleotide polymorphisms (SNP) in genes regulating pro-inflammatory cytokines, and patient outcomes (Zeiler et al., 2019e; Zeiler et al., 2019f). Genetic variation in immune response has also been highlighted as an important consideration in the genetics of biological aging, where SNP’s in IL-6 has been underlined as an important future area to consider in aging research (Cardoso et al., 2018).

Finally, neural modulation and repair mechanisms are also impaired as a result of aging. Decreased expression of brain derived neurotropic factor (BDNF) has been recently associated with advancing age. SNP’s in BDNF have also received note as potential areas of importance for future research in aging (Cardoso et al., 2018). Of note, BDNF SNP’s have also been linked to long-term outcomes in adult TBI literature (Zeiler et al., 2019e; Zeiler et al., 2019f). It may be that specific BDNF SNP’s potentiate impaired BDNF expression associated with aging, which leads to reduction in downstream regenerative mechanisms post-injury. Such impaired recovery mechanisms in the central nervous system may lead to increase in the pro-inflammatory process described previously, leading to worse cerebral vascular function.

### Sex as a Biological Consideration

As an additional layer of complexity, biological sex has some potentially major roles to play in cerebral vascular responses both in healthy and disease states. It impacts cerebrovascular responses both independent of, but also potentially mixed effects with, advancing age. With respect to moderate/severe TBI, the incidence of such injury overwhelmingly favors males. The majority of studies to date on TBI have had male predominance in their cohorts, limiting proper assessment of the impact of sex on secondary injury patterns and outcome (Maas et al., 2017; Steyerberg et al., 2019). This is also the case with all of the above-mentioned studies on MMM and their association with biological sex. However, biologically, sex differences leave important considerations regarding disparities in treatment responses, secondary injury severity, and outcomes (Mikolić et al., 2021).

With regards to progesterone in moderate/severe TBI, as mentioned in the introduction, substantial prior work has been conducted. Pre-clinical models of TBI have provided evidence that progesterone carries a neuroprotective effect, with exogenous supplementation in animals leading to reduced neural loss, improved neurophysiologic and functional outcomes (Clevenger et al., 2018; Khaksari et al., 2018). In humans, sex-related disparities in outcome after moderate/severe TBI have been well documented, despite the overwhelming majority of patients being male (Maas et al., 2017; Steyerberg et al., 2019; Mikolić et al., 2021). Supplementation with progesterone has been explored in such TBI cohorts in numerous randomized control trials evaluating the impact on long-term global outcomes, including the Progesterone for Traumatic Brain Injury, Experimental Clinical Treatment (ProTECT) (Wright et al., 2007402) and Efficacy and Safety Study of Intravenous Progesterone in Patients With Severe Traumatic Brain Injury (SyNAPSe) Trials (Skolnick et al., 2014). However, despite early evidence suggesting a potential impact on mortality and functional outcome (Pan et al., 2019), all trials have failed to demonstrate long-term impacts on mortality and morbidity (Lu et al., 2016; Ma et al., 2016; Schumacher et al., 2016; Pan et al., 2019). Though, functional outcomes assessments in trials were limited to course outcomes metrics, standard in moderate/severe TBI, such as the Glasgow Outcome Scale (Lu et al., 2016; Ma et al., 2016; Pan et al., 2019). Similarly, progesterone supplementation has also failed to lead to reproducible measurable differences in biomarkers of neural injury in such populations (Korley et al., 2021). To date no assessments of sex steroid supplementation and its impact on high-frequency cerebral physiology in adult moderate/severe TBI have been conducted.

Aside from progesterone, circulating estrogen levels have a well-documented impact on cerebral vasculature (Krause et al., 1985). Estrogen is known to promote prostaglandin I2 (PGI2) release, an important vasodilator (Krause et al., 1985; Deer and Stallone, 2014). Similarly, in males, or instances of low estrogen production, thromboxane A2 (TXA2) predominates, leading to cerebral vasoconstriction and impaired vasodilatory responses of the cerebral vessels (Deer and Stallone, 2014). Furthermore, in young female mice with higher circulating estrogen levels, there appears to be an attenuated angiotensin II/ROS response, with reduced vasoconstriction responses seen in female models to ROS exposures (De Silva et al., 2009). Finally, experimental literature points to high endothelial nitric oxide synthase (eNOS) vasodilation in female versus male rats, under exercise conditions (Arrick et al., 2016).

Aside from the endothelial and myogenic impacts of estrogen on the cerebral vasculature, there is a known anti-inflammatory effect. Data suggests that higher circulating estrogen levels are linked to decrease in leukocyte adhesion to the cerebral vasculature (Krause et al., 1985). Furthermore, estrogen has been shown to decrease inducible nitric oxide synthase (iNOS) in response to IL-1B, which is linked to upregulation of cyclooxygenase-2 (COX-2) and prostaglandin E2 (PGE2). As COX-2 and PGE2 are known cerebral vasoconstrictors, circulating estrogen levels subsequently attenuate their responses in the setting of injury (Krause et al., 1985). These anti-inflammatory properties of estrogen have triggered neuroprotective studies in both experimental and human settings (Brotfain et al., 2016; Späni et al., 2018; Martin-Jiménez et al., 2019; Yilmaz et al., 2019). However, their impact on global outcome post-TBI in humans is still uncertain.

Finally, aside from estrogenic endothelial/myogenic and anti-inflammatory effects, there have been documented differences in potassium channel expression in males vs. females, with a direct link to estrogen levels (Reed et al., 2020). In rats, large potassium channel beta-1 subunit (BK-B1) displays decreased expression in both males and female rats with removed ovaries. This BK-B1 reduction leads to increased MCA reactivity, as the large potassium channels are known to regulate cerebrovascular myogenic activity. This has yet to be substantiated in humans, but carries potential to explain discrepancies in cerebrovascular physiology seen in health and disease.

### Potential Mixed Biological Effects of Aging and Sex

Accounting of the biological impacts of aging and sex highlighted above, it’s clear that there is a potential for interaction on cerebrovascular function in the setting of TBI. This is particularly the case for aging females (Krause et al., 1985). During youth, females do benefit from the protective effects of higher circulating estrogen on cerebral vasculature. However, during menopause or post oophorectomy the risk profile may change. Menopause in particular, occurring with advancing age may now dramatically increase the risk of abnormal vascular responses in the setting of TBI. Such changes may be reflected in the emerging literature suggesting worse long-term outcomes for females suffering from TBI. It must be noted that in reviewing the above-outlined literature on MMM and its association between age and biological sex, there were no studies identified which adjusted for both age and sex in a multi-variable model. This is despite having age and sex information available in their core data sets. As such, it is clear that such future works in this area will need to evaluate such age/sex interactions in a more comprehensive manner.

### Future Directions

As outlined in the review on MMM provided and overview of the cerebrovascular biological considerations of aging and sex, there are some key areas for future investigation. First, the initial future works on MMM and its links with age and sex, require the use of high-resolution measures (Smieleweski and Czosnyka, 2000-2021). Relying on grand mean summary metrics for MMM physiology is insufficient, as signal variance is essentially eliminated, and the ability to discern sub-classes based on age and sex near impossible. As such, future works in the area require the use of time-series methodologies (Thelin et al., 2019; Zeiler et al., 2020e; Zeiler et al., 2020f), including autoregressive integrative moving average (ARIMA) and vector autoregressive integrative moving average (VARIMA) techniques. These should be applied based on large populations of TBI patients with high-frequency physiology, using sub-groups of age and sex. Further, sub-class evaluation can take place using semi-supervised or unsupervised machine learning techniques, and latent class analytics. Furthermore, more complex multi-variable models adjusting for age and sex are needed, as such literature is lacking. Such analyses may facilitate answering some of the preliminary questions regarding age/sex interactions, which currently remain unanswered in moderate/severe TBI. This work is the focus of various multi-center groups focused on high-frequency physiology data processing in both Europe (Howells et al., 2012; Maas et al., 2015; Güiza et al., 2017; Bennis et al., 2020) and Canada (Zeiler et al., 2018d; Bernard et al., 2020), and requires multi-disciplinary integrated teams of clinicians, engineers, physiologists and data scientists.

Aside from the initial more detailed analytic pathways for MMM physiology, the second phase of such inquiry necessitates integration of the physiome data with proteomic and genomic information. In general, genome wide association data (Zeiler et al., 2019e; Zeiler et al., 2019f), combined with proteomic information (Timofeev et al., 2011; Maas et al., 2015; Thelin et al., 2017b), may shed light on particular pathways driving cerebral physiologic dysfunction in TBI. However, with respect to aging and sex, integrating blood/serum/CSF/microdialysate sampling during the acute phase of TBI care, with high-resolution MMM data is key. First, blood can be utilized for genome wide association studies (GWAS), identifying various SNP’s of interest, including those related to pro-inflammatory cytokines, neural repair and angiotensin pathways that are of interest in advanced age (Cardoso et al., 2018; Zeiler et al., 2019f). Second, serum/CSF/microdialysate can be exploited to quantify circulating levels of cytokines (Helmy et al., 2011a; Helmy et al., 2011b; Zeiler et al., 2017c; Thelin et al., 2018), such as IL-6, nitric oxide synthase activity (through nitrate/nitrite levels), prostaglandin levels, free radical analysis, and assessment of sex hormone concentrations. Finally, blood can also be exploited to provide proper quantification of biological age, using current epigenetic techniques (Ishikawa et al., 2016; Bisset and Howlett, 2019; Zarzour et al., 2019). It is well known the chronological age is a poor marker of biological age and function. Epigenetic techniques evaluating widespread genome methylation status, histone modifications, or telomere length of circulating white cells, can be used to quantify biological age. Such epigenetic information may allow us to more accurately determine biological age and its link to MMM based physiologic dysfunction in TBI. However, such work is easy to describe, but much more complicated to execute. As with the advanced signal analytics, such work requires large multi-center data sets for reasons of statistical power and multi-disciplinary expertise. Some preliminary work in this area is being done using the CENTER-TBI high-resolution data set (Maas et al., 2015), but will only set the stage for future larger multi-center works in the area of advanced omics research in TBI.

## Conclusion

The current literature body on the association between age and sex with high-frequency continuous MMM based cerebral physiological measures in moderate/severe TBI is lacking. Most studies use coarse summary metrics, documenting association between physiology and chronological age and/or binary sex designations. Few studies to date account for biological processes of aging and sex, and their impact on MMM physiology. Future work on the impact of aging and sex on MMM physiology in TBI will require advanced signal analytic techniques, with integrated proteomic and genomic data, while accounting for biological age using epigenetics.
